# Analysis of Environmental Stress Factors Using an Artificial Growth System and Plant Fitness Optimization

**DOI:** 10.1155/2015/292543

**Published:** 2015-03-22

**Authors:** Meonghun Lee, Hyun Yoe

**Affiliations:** Department of Information and Communication Engineering, Sunchon National University, Jeonnam 540-950, Republic of Korea

## Abstract

The environment promotes evolution. Evolutionary processes represent environmental adaptations over long time scales; evolution of crop genomes is not inducible within the relatively short time span of a human generation. Extreme environmental conditions can accelerate evolution, but such conditions are often stress inducing and disruptive. Artificial growth systems can be used to induce and select genomic variation by changing external environmental conditions, thus, accelerating evolution. By using cloud computing and big-data analysis, we analyzed environmental stress factors for *Pleurotus ostreatus* by assessing, evaluating, and predicting information of the growth environment. Through the indexing of environmental stress, the growth environment can be precisely controlled and developed into a technology for improving crop quality and production.

## 1. Introduction

Plant growth is a multifaceted process that integrates physiological phenomena such as photosynthetic activity, water use, nutrient uptake, storage of starch and lipids, and adaptation to stress [1]. In contrast to animals, plants cannot relocate to avoid stress, and thus a sophisticated internal control system is needed to combat adverse conditions [2]. Research on plant adaptations to stress should focus on understanding the overall system and its complexity. All plants experience some environmental stress, which, in the absence of adaptation, can lead to reduced resistance to pests and to disease.

Representative examples of environmental stressors experienced by plants can include drought or excess moisture, poor aeration, lack of sunlight, and high or low temperature. The optimal plant growth environment should be provided by identifying early stages of biological reactions to stressors through frequent observation [3]. Symptoms of pest-related damage should also be assessed at an early stage through observation of leaves and branches. Early diagnosis and treatment can reduce damage caused by disease.

Artificial growth systems (e.g., factory farms) are production systems that can grow crops year-round by artificially adjusting the natural environment (e.g., light, temperature, humidity, carbon dioxide, airflow, and nutrients) within confined facilities [4]. Optimization of plant fitness through control of the growth environment enables quality and yield to be improved. Manipulation of growth environmental conditions can also accelerate evolution by inducing and selecting genomic variation.

Here, we report the use of a data-intensive cloud service to store, process, and analyze environmental information generated from an artificial growth system [5]. The importance of cloud computing in the field of bioinformatics is growing rapidly as large-scale biological and environmental datasets become available [6–8]. The purpose of data-intensive computing is to find biological knowledge by analyzing big data; the data parallelism technique is the primary processing method and consists of calculations that are assisted by cloud computing. Data-intensive computing uses advanced technology that can handle high-capacity searches and data extraction [9, 10]. The artificial growth system implemented here is an integrated system that can analyze patterns in biological big data and process growth environmental data quickly and continuously.

To implement this system, this study is organized as follows.  Section 2 examines the broad configuration of the artificial growth system developed in this study; Sections 3 and 4 provide a detailed discussion of cloud service and Internet-of-Things (IoT) devices for cloud or artificial growth systems.  Section 5 analyzes environmental stress factors for* Pleurotus ostreatus* through a pilot project. Finally, Section 6 presents conclusions and directions for future research.

## 2. Artificial Growth System

**Figure 1:**
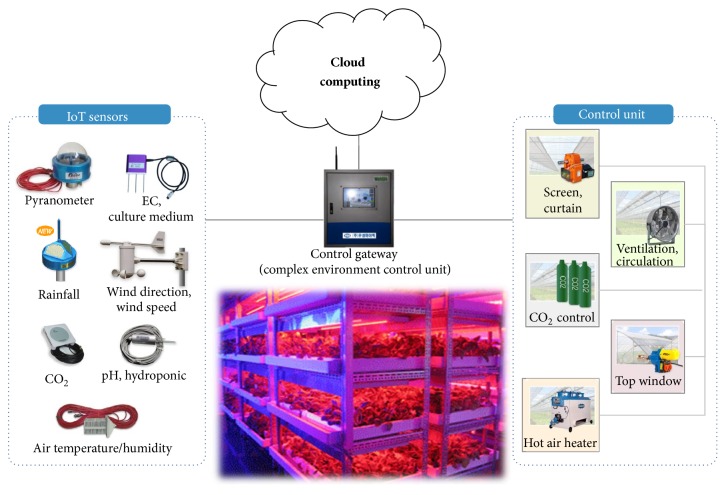
Schematic diagram of an artificial growth system.

The purpose of protected cultivation is to harvest plants at the desired time by conversion from passive crop production, which relies on natural conditions, to active (“artificial”) production [11]. In this study, “plant” refers to the entire plant body, including tissues and cells; the plant is referred to as an artificial growth system, a clonal proliferation system, or a cell mass-culture system, depending on the production system used. In advanced facilities such as artificial growth systems, genetic variation can be induced because most environmental factors relating to crop growth are tightly controlled [12]. To operate the artificial growth system, as shown in Figure 1, an environmental control unit is constructed using elements such as light-emitting diodes (LEDs) and temperature, humidity, and carbon dioxide (CO_2_) regulators.

*(1) IoT Sensors*. The artificial environment is controlled by monitoring plant growth status using sensors for electrical conductivity (EC), pH, temperature, humidity, CO_2_, and light [13]. Basically, growth-related sensors require EC and pH sensors, and temperature, humidity, CO_2_, and pyranometer sensors are used as the environment-related sensors. These sensors enable the real-time growth environment of plants and roots to be monitored.

*(2) Control Units*. The artificial growth system includes means to control various facilities within the system to maintain optimal conditions for crop growth. The control units are based on information collected using the sensors and are designed to be used remotely via a communication interface such as the Internet.

If sensor data are entered, which are outside of the previously established acceptable range for the facility, the facility can be controlled in real time through a control-status lookup feature. This design enables more precise control, through IoT technology, than is possible with traditional wired systems.

*(3) Control Gateway*. Information collected from the system by sensors in real time is stored in a cloud database (DB) that can be accessed by the user at any time through a network. The data analysis provides information (e.g., external weather data, heat, and water status) needed for facility control and management.

## 3. Data-Intensive Cloud Service

**Figure 2:**
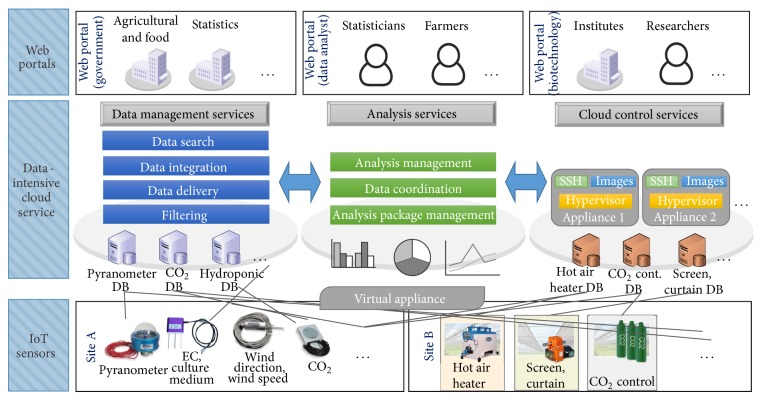
Data-intensive cloud service platform.

The types of platforms and computing resources required for data analysis are very diverse. As shown in Figure 2, the data-intensive cloud service platform consists of analysis services based on virtual infrastructure and data management that enables efficient access to high-capacity data. We created a virtual machine (VM) to logically control, integrate, and manage various IoT sensors and applications for growth environment control through analysis of environmental data and implementation of an IoT-centric cloud using a virtual appliance (VA) equipped with each sensor application.

*(1) Data Management Services*. Data management services allow users to search and download remote environmental data through web-portal interfaces. Searches can be performed using actual data values or metadata as a property and data that are necessary for the analysis can be selectively extracted. Data are sent to the user in an efficient and transparent manner. These services are provided via a user portal developed separately to optimize user convenience and accessibility.

The information collected from IoT sensors includes structured and unstructured data that must be filtered to enable effective utilization. Data filtering includes four steps: parsing for discovery of formatting and other types of errors; transformation to fit specific formats; elimination of duplicates; and statistical correction of missing or erroneous data. 

*(2) Analysis Services*. Analysis services create virtual instances that perform data analysis according to specifications required by the user. The virtual instance is one independent computing cluster composed of a virtual machine for data analysis, with a system configured for data access and task management. Virtual instances are created and managed through virtual cluster services. Analysis services can select and implement virtual cluster appliances or directly register and use new virtual appliances.

*(3) Virtual Appliance*. The VA is a plug-in that allows the user to quickly build an artificial growth system. The VA can remotely control storage, deletion, and modification of data in the cloud service in image form; it sends and receives necessary data through the cloud-computing server interface and enables the VM to be managed through synchronization with the cloud. The advantage of the VA is excellent mobility; it can be installed without limitation if a network connection is available.

If the Linux-based Hypervisor is installed and embedded, the VM can be operated in the environment [14]. Hypervisor can be configured with Xen or Kernel-based virtual machines (KVM) [15]; a VM is produced which minimizes the appliance load in the embedded form, with an optimal environment that can run applications and an adapter that enables communication among sensors. Each VM has a DB that is used to analyze data and control its sensors. IoT sensor-control applications installed on the VM are used for self-analysis and control of collected data; some data are transmitted to data management centers in the cloud service and used for statistical analyses, control, and linkage to other systems.

*(4) Cloud Control Services*. Cloud control services are used to monitor and control sensors by analyzing the data collected by IoT sensors. These services configure VAs into clusters and monitor the status (including memory and available capacity) of each cluster node, VM, CPU, and memory information and available capacity. Various applications for controlling the sensor are included.

**Figure 3:**
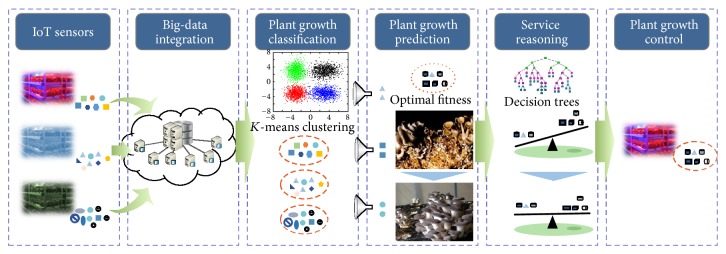
Plant fitness optimization in cloud computing.

*(5) Data Mining Techniques*. The process by which analytical results are obtained from environmental big data is illustrated in Figure 3. Optimization of plant fitness involves six steps: data collection, storage, analysis, prediction, evaluation of results, and application.

The artificial growth system was set up to handle all requests and to establish an integrated solution for the process that tightly unites all stages of the application. An exploratory data analysis (EDA) methodology was used to extract meaningful data, and the structure of the environmental mutation data was identified through mutagenesis cluster analysis, which used the *K*-means algorithm to classify specimens that had undergone ecological mutations into clusters [16]. Those specimens that underwent mutagenesis attributed to environmental factors were classified into groups and the properties of each group were determined to gain an understanding of all of the target specimens.

## 4. Plant Fitness Optimization in Cloud Computing

The artificial growth system is analyzed based on historical information regarding sowing, plant growth conditions, and harvest; appropriate conditions are then defined based on this analysis. Appropriate growth conditions should be determined by intelligently predicting changes in growth stage from these data and assessing the results of those predictions on the controlled growth system. These processes are illustrated in Figure 3.

Growth information is divided into external and internal data. External data include the temperature of the culture medium, CO_2_, humidity, and light conditions in the artificial growth system; internal data include the historical DB in which plant growth status is recorded. External growth information collectors retrieve data in real time using IoT sensors, and internal growth information collectors save growth status data in real time in the historical DB and transmit these data to a big-data integrator. Big-data integration processes and saves the data for use in plant growth classification.

Plant growth classification categorizes and disassembles datasets with *K*-means clustering. The plant fitness optimization clustering technique clusters the related growth information through similarity of the information, thus helping to classify, search, and process the large amount of information automatically.

Plant growth prediction determines the time at which the growth stage changes by predicting changes in fresh weight from one growth stage to the next, according to the classified growth conditions. Service reasoning uses proposed reasoning rules to provide conditions that are suitable for a given growth stage at the time of the predicted change in growth stage.

## 5. Analysis of Environmental Stress Factors

Plant biotechnology has advanced, with traditional plant-breeding techniques used to design and produce crops that suit human requirements. New convergence studies involving crop molecular systems and biological and human evolutionary studies have emerged as a new research paradigm [17]. Big-data analysis combined with IoT and establishment of year-round crop-producing systems are being attempted for the first time for* Pleurotus ostreatus*.

Through a pilot project in which* P. ostreatus* was cultivated in an artificial growth system, we performed a rapid analysis of large amounts of environmental data and analyzed environmental stress factors using distributed computing technology. This work has generated a database of biological information for fungi that can continue to be expanded. Characteristics of* P. ostreatus* change according to growing conditions [18]; these morphological changes can be measured in relation to symptoms of physiological disorder that result from failure of the environmental control system.

*(1) Fruiting Body under High Temperature and High Humidity*. If the temperature and humidity in the artificial growth system exceed 20°C and 80%, respectively, the fruiting body of* P*.* ostreatus* is relatively short compared to the stalk, and the color ranges from light gray to grayish brown. Some mushrooms project a pileus center that appears as a small lump, whereas others have a central depression (dimple).

*(2) Fruiting Body under High Temperature and Low Humidity*. If the temperature in the artificial growth system exceeds 20°C and the humidity is 80% or lower, the pileus edge of most* P*.* ostreatus* becomes thin, and the mushroom becomes umbrella-shaped with light gray or white color. The mushroom's fresh weight decreases significantly and the fruiting body can be easily broken during harvest. In addition, the stalk becomes thin, and malformed mushrooms with inconsistent pileus and indistinct stalks occur frequently.

*(3) Fruiting Body under Low Temperature and Low Humidity*. The most-pronounced change in the* P*.* ostreatus* fruiting body at temperatures <12°C is that the flesh becomes dark brown and acquires a very hard texture. Mushroom growth is very slow and bacterial blotches can appear, and the pileus widens or becomes rough and bent. The stalk becomes thick and jar-shaped, and the central region can become enlarged. The germination rate of the mushrooms is significantly reduced, which affects yield.

*(4) Fruiting Body under Low Temperature and High Humidity*. At low temperatures (13–16°C) and high humidity (>80%), the quality of* P*.* ostreatus* is good; the flesh is dark brown and firm, and the pileus is thick. However, mushroom growth and germination rates are poor compared to normal temperatures.

*(5) Fruiting Body under Optimum Temperature and Humidity*. Cultivation of* P. ostreatus* at 13–16°C and humidity >80% can improve physiological disorders in this species.

If only a subset of environmental factors is managed, problems will arise in the artificial growth environment. When a balance is maintained among the environmental conditions, these factors can either complement or oppose one another. If temperature increases, humidity decreases; if ventilation is supplied when the air temperature is higher than the temperature in the artificial growth system, the temperature increases and humidity decreases. In spring and autumn, proper ventilation maintains the temperature and generates optimal growing conditions. In spring and autumn cultivation, the temperature does not require manipulation because the external temperature is ideal for mushroom growth. However, growers should be careful regarding humidity and ventilation at this time.

Additionally, we found that the CO_2_ concentration in the artificial growth system was closely related to mushroom growth. Excessive CO_2_ during growth and ripening was the main cause of malformed mushrooms.

Morphological changes in* P. ostreatus* were examined in relation to CO_2_ concentration. As the CO_2_ concentration increased, the size of the pileus decreased and stalk length increased. The mushroom fresh weight was highest in the 0.03% CO_2_ treatment group (342 g per 1,100 mL bottle) and decreased with increasing concentration (Table 1).

## 6. Conclusions

Artificial growth systems enable planned production of crops through strict environmental control. Plants survive by adapting appropriately to changing environmental conditions, but on exposure to external stressors, reactive oxygen species levels increase and damage plant cells. As one defense mechanism, plants produce functional materials with antioxidant properties. In the artificial growth system, functional material-centered qualities can be improved by using short-duration environmental stress.

**Figure 4:**
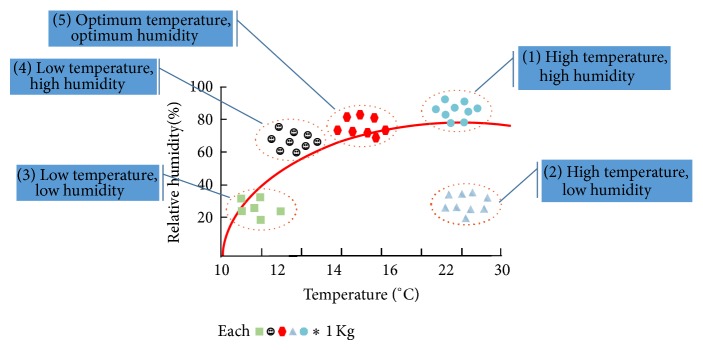
Comparison of physiological characteristics of* Pleurotus ostreatus* grown in different culture conditions.

As shown in Figure 4 and Table 1, several genetic events, which can be influenced significantly by the surrounding environment, need to occur to produce a high yield and quality of* P. ostreatus* fruiting bodies. The three conditions that must be controlled are temperature, humidity, and ventilation; if these conditions are not adequately managed, all variants will experience reduced quality and physiological disorder.

In the present study, collection, storage, processing, and analysis of big-data-based biological and environmental information were implemented using cloud computing. In addition, a cloud service that can be easily used by both biologists and bioinformatics experts was presented. To efficiently analyze patterns in biological and environmental data and to implement an integrated system that can use a database based on distributed cloud computing, we designed all necessary modules in each component. Our artificial growth system analyzed biological growth patterns and focused on optimizing fitness. In conclusion, by indexing environmental stressors, the growth environment can be precisely controlled and developed into technology for improving crop quality and yield.

## Figures and Tables

**Table 1:** CO_2_ concentration for fruit-body formation and yield of *Pleurotus ostreatus* in the artificial growth system.

Item	CO_2_ concentration (%)			
	0.03	0.15	0.32	0.53
Pileus (mm)	6.4	3.2	2.6	0.7
Stalk (mm)	4.9	6.5	6.4	2.3
Fresh weight (g/bottle)	342	245	197	95

## References

[1] Kim Y. C., Leveau J., Gardener B. B. M., Pierson E. A., Pierson L. S., Ryu C.-M. (2011). The multifactorial basis for plant health promotion by plant-associated bacteria. *Applied and Environmental Microbiology*.

[2] Bohnert H. J., Gong Q., Li P., Ma S. (2006). Unraveling abiotic stress tolerance mechanisms—getting genomics going. *Current Opinion in Plant Biology*.

[3] Sreenivasulu N., Sunkar R., Wobus U., Strickert M., Sunkar R. (2010). Array platforms and bioinformatics tools for the analysis of plant transcriptome in response to abiotic stress. *Plant Stress Tolerance*.

[4] Sivamani S., Bae N., Cho Y. (2013). A smart service model based on ubiquitous sensor networks using vertical farm ontology. *International Journal of Distributed Sensor Networks*.

[5] Gorton I., Greenfield P., Szalay A., Williams R. (2008). Data-intensive computing in the 21st century. *Computer*.

[6] Edwards D., Batley J. (2004). Plant bioinformatics: from genome to phenome. *Trends in Biotechnology*.

[7] Rosenberg N. J. (1983). *Microclimate: The Biological Environment*.

[8] Gunarathne T., Wu T.-L., Choi J. Y., Bae S.-H., Qiu J. (2011). Cloud computing paradigms for pleasingly parallel biomedical applications. *Concurrency Computation Practice and Experience*.

[9] Issa S. A., Kienzler R., El-Kalioby M. (2013). Streaming support for data intensive cloud-based sequence analysis. *BioMed Research International*.

[10] Lin Y.-C., Yu C.-S., Lin Y.-J. (2013). Enabling large-scale biomedical analysis in the cloud. *BioMed Research International*.

[11] Hussey N. W., Read W. H., Hesling J. J. (1969). *The Pests of Protected Cultivation. The Biology and Control of Glasshouse and Mushroom Pests*.

[12] Jiang W., Qu D., Mu D., Wang L. (2004). Protected cultivation of horticultural crops in China. *Horticultural Reviews*.

[13] Swan M. (2012). Sensor mania! the internet of things, wearable computing, objective metrics, and the quantified self 2.0. *Journal of Sensor and Actuator Networks*.

[14] Hariprasad G. (2013). *Porting Linux to a Hypervisor Based Embedded System*.

[15] http://www.xenproject.org/.

[16] James G., Witten D., Hastie T., Tibshirani R. (2013). *An Introduction to Statistical Learning: with Applications in R*.

[17] Ma C., Zhang H. H., Wang X. (2014). Machine learning for Big Data analytics in plants. *Trends in Plant Science*.

[18] Rural Development Administration (2004). *Oyster Mushroom*.

